# The Synergistic Effect of Biosynthesized Silver Nanoparticles and Phage ZCSE2 as a Novel Approach to Combat Multidrug-Resistant *Salmonella enterica*

**DOI:** 10.3390/antibiotics10060678

**Published:** 2021-06-05

**Authors:** Abdallah S. Abdelsattar, Rana Nofal, Salsabil Makky, Anan Safwat, Amera Taha, Ayman El-Shibiny

**Affiliations:** 1 Center for Microbiology and Phage Therapy, Zewail City of Science and Technology, Giza 12578, Egypt; p-abdallah.abdelsattar@zewailcity.edu.eg (A.S.A.); p-rnofal@zewailcity.edu.eg (R.N.); syoussef@zewailcity.edu.eg (S.M.); agaber@zewailcity.edu.eg (A.S.); s-ameraelsayed@zewailcity.edu.eg (A.T.); 2 Center for X-ray and Determination of Structure of Matter, Zewail City of Science and Technology, Giza 12578, Egypt; 3 Faculty of Environmental Agricultural Sciences, Arish University, Arish 45511, Egypt

**Keywords:** AgNPs, antibacterial, antimicrobial, antibiotic-resistant bacteria, green synthesis, phage therapy, green synthesis, synergetic effect, FTIR, time-killing curve

## Abstract

The emergence and evolution of antibiotic-resistant bacteria is considered a public health concern. *Salmonella* is one of the most common pathogens that cause high mortality and morbidity rates in humans, animals, and poultry annually. In this work, we developed a combination of silver nanoparticles (AgNPs) with bacteriophage (phage) as an antimicrobial agent to control microbial growth. The synthesized AgNPs with propolis were characterized by testing their color change from transparent to deep brown by transmission electron microscopy (TEM) and Fourier-Transform Infrared Spectroscopy (FTIR). The phage ZCSE2 was found to be stable when combined with AgNPs. Both minimum inhibitory concentration (MIC) and minimum bactericidal concentration (MBC) were evaluated for AgNPs, phage, and their combination. The results indicated that MIC and MBC values were equal to 23 µg/mL against *Salmonella* bacteria at a concentration of 10^7^ CFU/mL. The combination of 0.4× MIC from AgNPs and phage with Multiplicity of Infection (MOI) 0.1 showed an inhibitory effect. This combination of AgNPs and phage offers a prospect of nanoparticles with significantly enhanced antibacterial properties and therapeutic performance.

## 1. Introduction

*Salmonella* is one of the most common pathogens with transfer occuring from animal feces to food, soil, and water. It is considered the second most frequently reported pathogen that is associated with zoonosis. As a facultative anaerobic Gram-negative, non-spore-forming, and non-capsulated bacteria, *Salmonella* belongs to the *Enterobacteriaceae* family [1]. It is responsible for around 150,000 deaths annually. About 93 million cases are diagnosed yearly with salmonellosis, accompanied by symptoms of gastroenteritis, bacteremia, and enteric fever [2]. Antibiotics are still considered the first line of treatment of *Salmonella* infection, including ampicillin, amoxicillin, chloramphenicol, and trimethoprim-sulfamethoxazole. However, due to excessive use of antibiotics in therapeutic and industrial applications, multidrug-resistant (MRD) *Salmonella* is widely spread and transmitted from animals to humans [3]. Following the spread of antibiotic-resistant strains, alternatives are sought to limit the resulting medical and economic effects.

Phages are among the promising alternative to antibiotics. Virulent phages are viruses that infect bacterial cells, produce new virions, and obligatorily kill their hosts. Phages are the natural enemies of bacteria, without interfering with mammalian and human cells [4,5]. Their narrow host range enables their specific targeting of bacterial species or sub-strains in the same species, including *Salmonella* treatment [6,7]. Compared to antibiotics, phage isolation and laboratory preparation are time, effort, and cost-effective, yet further efforts are needed to transfer phage applications from laboratory bench to markets and to increase their shelf life [8,9,10]. However, the issue of bacterial resistance development towards phages could happen during the process of phage treatment [11,12,13,14]. Therefore, to improve phage efficiency, tolerance, and delivery, recent approaches support coupling phages with other bio-control agents such as antibiotics [15], natural products (e.g., venom, propolis, and extracted oils) [16], phage purified enzymes (e.g., lysins, endopeptidases, amidases, and transglycosylases) [17], in addition to syntactic compounds and nanoparticles [18,19,20].

Another approach is to use antibacterial compounds such as silver, which has been administrated before as an antimicrobial agent due to its efficiency against Gram-positive and Gram-negative bacteria [21,22]. AgNPs have growth inhibitory effects on various multidrug-resistant bacteria including *Salmonella* [23,24]. Similar to phages, the antimicrobial action of AgNPs relies on the recognition of the bacterial cell wall and membrane, bacterial penetration and damage through inducing the cellular toxicity and oxidative stress [25]. However, many studies have revealed various concerns regarding unpleasant side effects of AgNPs on human internal organs such as the lung, liver, and neurons [26,27]. Therefore, it is recommended to apply low concentrations of AgNPs to limit the side effects on human health and the environment. One common method is to use natural compounds such as an extract of propolis as a capping agent for AgNPs [28,29]. Propolis is produced by honeybees and is rich in flavonoids and phenolic acids [30]. In addition, it has many biological benefits including antimicrobial activity, antioxidant effect, in addition to its ability to improve the immune function [31,32].

Few studies have investigated the effect of combining the two approaches: phages with metal nanoparticles. The previous findings were controversial since they reported that metal nanoparticles such as silver, gold, and copper oxide inactivate T4 phage, indicating that phages are not stable when combined with nanoparticles [33]. Another study suggested that AgNPs negatively affected a phage’s life cycle because the nanoparticles interfere with the bacterial host cell and respectively might weaken the infectivity of phages [34]. Whereas, other studies introduced the combined approach, of C3 phage and gold nanoparticles (AuNPs), as a promising treatment for *Pseudomonas aeruginosa* planktonic and biofilm states, with high stability under a broad range of temperature, pH, and salt concentration [35]. Moreover, other recent research work highlighted the high potential of green AuNPs and phage combination in eradicating the multi-drug resistant *Staphylococcus aureus* biofilms [36]. These data represent a gap in understanding the underlying of mechanism and effect of the phage-NPs combined approach.

Although both phages and AgNPs independently present interesting provisionary antibacterial implications, they have some limitations. Herein, we hypothesize that by mixing phage with AgNPs coated with propolis as a natural product, we can ascertain their bio-control capabilities with low doses of AgNPs and low incidences of resistance. Accordingly, this study amalgamates the activity of the previously isolated virulent phage ZCSE2 with a freshly-made and well-characterized AgNPs to investigate the possible synergetic effect in controlling the growth and spread of *Salmonella*. The mixture of phage ZCSE2 and AgNPs as a treatment will enhance our understanding of phage-nanoparticle stability and interactions, in addition to providing a potential bio-control agent for various applications.

## 2. Results and Discussion

### 2.1. Characterization of AgNPs

**Figure 1:**
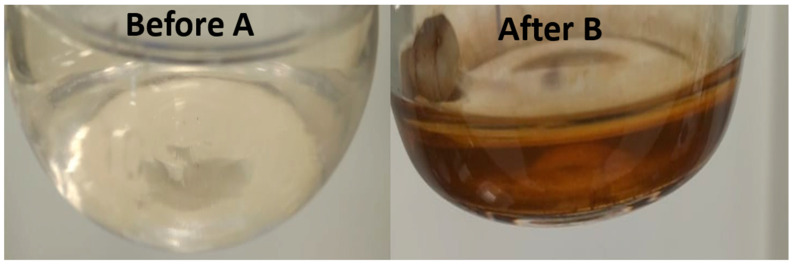
Flask A represents the color changes at time 0 (**before**) for AgNO_3_ and propolis extraction, while Flask B represents the biosynthesis of AgNPs following 5 h (**after**).

In this work, the bio-reduction of AgI ions to form the AgNPs was achieved successfully using the propolis extract presented as a bio-reducing agent. The AgNPs formation was confirmed by the color change of the transparent AgNO_3_ solution and propolis extraction into deep brown color after 5 h (Figure 1). AgNPs formation has also confirmed by the measurement of surface plasmon resonance in the resultant nanoparticles [37,38]. The various phytochemical compounds detected in the propolis extract (phenolic acids, flavonoids, and terpenoids) could be the reason behind the bio-reduction of the AgI ions and the capping with the formed AgNPs [39].

### 2.2. The UV–Vis Spectrum

**Figure 2:**
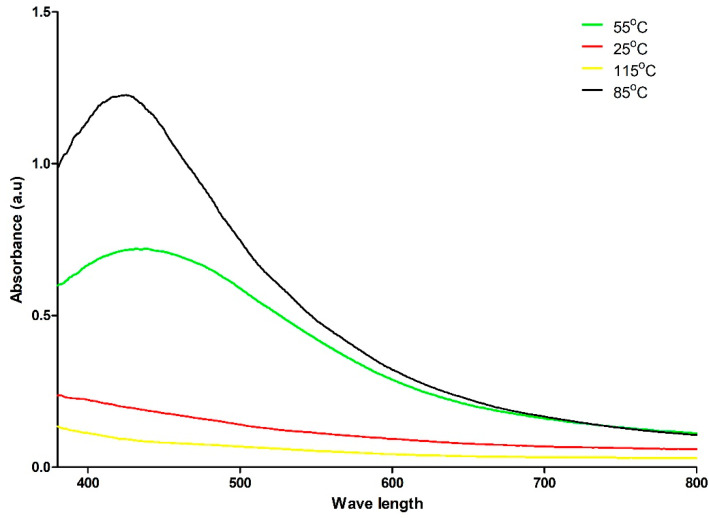
Shows the UV–Visible spectra of AgNPs coated by propolis extraction that were prepared at different temperatures.

The UV–Vis spectrum provided evidence of the formation of AgNPs prepared at 85 °C. The maximum absorption at 422 to 430 nm is an indication of surface plasmon resonance (Figure 2). By contrast, a single peak is visible at room temperature [40]. Bio-reduction of Ag^+^ through biomolecules found in the propolis could be the reason behind this observation [41]. In addition, the solution was diluted by using deionized water (Figure S6A). The resulting signal were time dependent (Figure S6B) and temperature dependent (Figure 2). The most optimal temperature, displaying a sharp peak at 422 to 430 nm was 85 °C, followed by 55 °C. However, the room temperature (25 °C) and above water boiling point (115 °C) preparations did not form nanoparticles. In previous work, the absorbance of biosynthesized AgNPs with propolis showed a similar wavelength peak from 324 to 449 nm and lower absorbance intensity ~2.25 a.u without dilution [29]. Another study supports λmax of AgNPs, which is biosynthesized by propolis extraction at 424 nm [28].

### 2.3. Visualization by TEM

**Figure 3:**
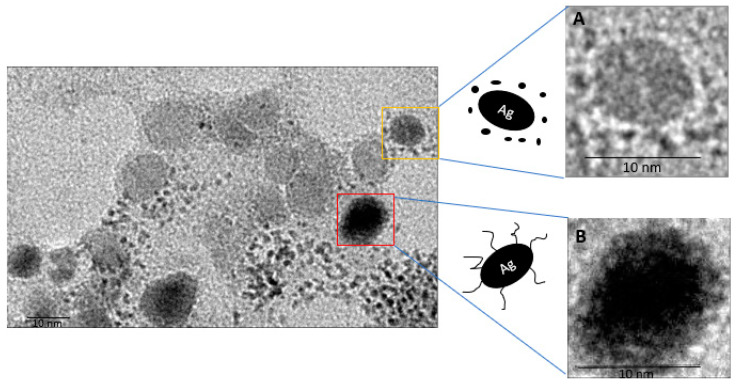
Presents the different sizes of AgNPs. (**A**) core-shell particles of AgNP. (**B**) AgNP coated with ethanolic extract of propolis. The scale bar is 10 nm.

**Figure 4:**
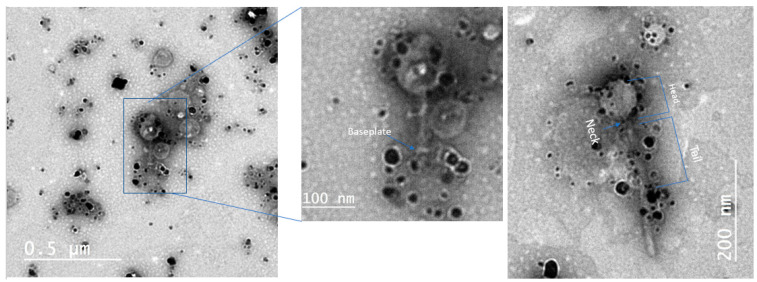
Shows the interaction between phage and biosynthesized AgNPs.

The morphology and size of the nanoparticles was examined by using TEM as shown in Figure 3. The micrographs showed that the biosynthesized AgNPs are oval in shape with a range of sizes from 2 to 41 nm in diameter, which is within the range of nanoparticles. Figure 3A represents the smart form of core-shell particles of AgNP and Figure 3B depicts the two different color intensities which indicate the capping process of AgNP with ethanolic extract of propolis [42]. This capping process is essential to control the size of AgNPs [43]. It enhances the antibacterial activity and biofilm clearance [44], provides bio-stability over the course of infection time [45], and most importantly it provides lower cytotoxicity effect [46]. It was clear that the AgNPs were bound to the capsid and tail fibers of the phage (Figure 4). This interaction will facilitate the delivery of nanoparticles during phage binding to specific bacterial receptors.

### 2.4. Zeta Potential

The zeta potential of the biosynthesized AgNPs displayed a sharp peak at −22.2 mV (Figure S4), which indicates that the surface of the AgNPs has a negative charge that is uniformly distributed in the solution. In addition, because of this negative charge the AgNPs showed that there was a high stability in the medium due to propolis extraction that electrostatically stabilized the biosynthesized AgNPs surfaces [47]. One of the important requirements to realize the use of AgNPs is to predict the interaction between nanoparticles and macromolecules in the body. For example, DNA carries a negative charge as AgNPs and this reduces the cytotoxicity of AgNPs in the cells [48]. Moreover, since most plasma proteins have a negative charge at physiological pH, the negative charge of AgNPs will provide a low risk of the protein corona [49].

### 2.5. FTIR Analysis

FTIR is one of the significant tools used to identify the functional groups and the presence of organic compounds that bind to biosynthesized AgNPs surfaces. The FTIR spectrum obtained from AgNPs coated by propolis extraction (Figure S5) displayed the following peaks 3385 cm^−1^ (for hydroxyls [50]), 2996 cm^−1^ (for stretching vibration (O)CH3 that found in propolis components [51,52], 2894 cm^−1^ for the saturated CH stretches), 1644 cm^−1^ (bonds in carbonyls and carboxy [53]), 1387 cm^−1^ (heterocyclic compounds (C–O–C) which is in alkaloids and flavones [54]), 1226 cm^−1^ (C–O group in hydroxyflavonoids [55]), and 1193 cm^−1^ (methylene beside the carbonyl group [56]). The FTIR analysis implied that the biological molecules had been involved in capping and stabilizing AgNPs.

### 2.6. Disc and Well Diffusion Methods

The antibacterial effect of the AgNPs was tested against pathogenic *S.* Enteritidis WT (Platten)*.* Direct spotting provides information on whether the concentration has an antibacterial effect or not. The diameters of the inhibitory zones of different concentrations of biosynthesized AgNPs were measured (Table 1). In our previous published work [6], the antibiotic sensitivity profile for the same strain was conducted and revealed that the bacterium is resistant to many antibiotics including Cefaclor 30 µg, Clarithromycin 15 µg, Erythromycin 15 µg, Vancomycin 30 µg, Linezolid 30 µg, and Novobiocin 30 µg [6]. However, AgNPs displayed a higher efficiency to inhibit the growth of the bacterial culture. All experiments were performed in parallel with the use of propolis extract to confirm that antibacterial activity is due to the biosynthesized AgNPs (Figure S3). AgNO_3_ solution had similar antibacterial activity to biosynthesized AgNPs against *Salmonella* bacteria when direct spotting, well, and disc diffusion methods are used (Table S2). AgNO_3_ is used as a supplier source of Ag^+^ in the solution because it has antibacterial activity against bacteria due to its ability to bind to biomacromolecules such as DNA, RNA, proteins [57,58]. The antibacterial effect of AgNPs is due to the inner membrane damage without affecting the outer membrane and the accumulation of reactive oxygen species and intracellular Ca^2+^ [59].

### 2.7. Phage Stability

**Figure 5:**
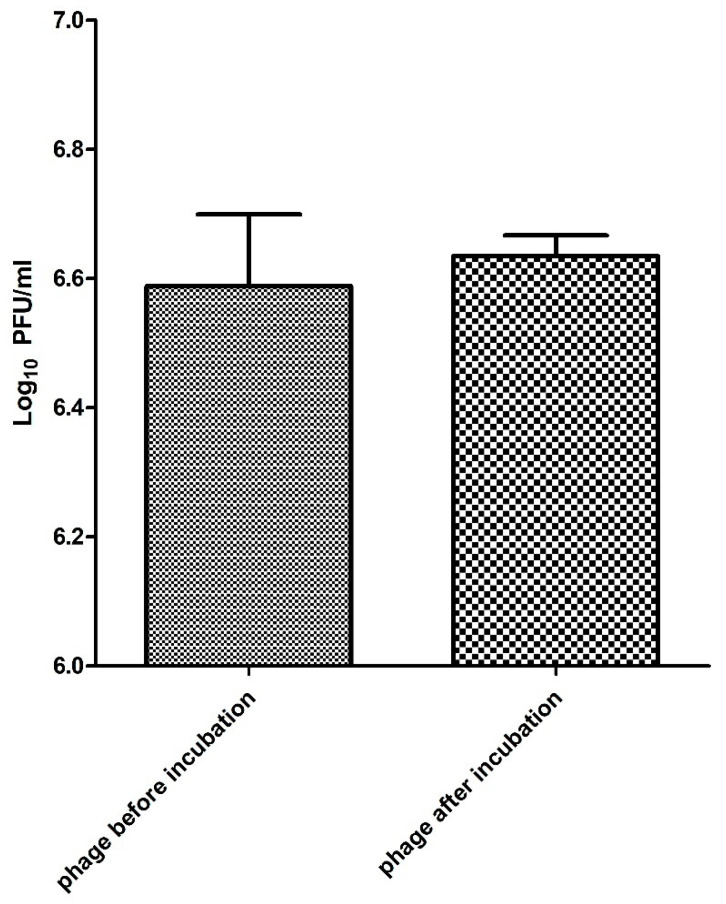
Displays the phage stability against AgNPs after incubation for 4 h.

The *Salmonella* phage ZCSE2 (MK673511) was characterized in order to determine its potential treatment to control the growth of *Salmonella.* The phage displayed high stability in different environmental conditions including pH and temperature [6]. To the best of our knowledge, no previous studies were conducted to investigate the stability of phage with biosynthesized AgNPs. This gap of information limits the possibility of combining phage therapy with antibacterial use of nanoparticles. Our results showed an in significant difference between the phage titer before and after incubation for 4 h with the 92 µg/mL of AgNPs at 37 °C (Figure 5). Compared to other viruses including (H3N2) influenza virus [60] and the chikungunya virus [61], phage ZCSE2 showed a higher stability when combined with AgNPs. The titer of ZCSE2 with AgNPs was around 3.8 × 10^6^ PFU/mL and the titer of phage alone was 2.1 × 10^6^ PFU/mL after 4 h at 37 °C without a significant difference (*p* > 0.05). This stability data cannot be generalized for all phage families since previous studies showed that high concentrations of AgNPs inactivated T4 phage [33]. Therefore, further work is needed to test the stabilities of different biosynthesized AgNPs on different phage families. Unfortunately, low-speed centrifugation and filtration can only remove some of the bacterial debris without reducing the lipopolysaccharides (LPS), peptidoglycan, and flagella [14] in addition to the different proteins that are produced as a result of bacterial burst after phage infection. This bacterial debris could interfere with AgNPs and affect their interaction with phage. For this reason, we used Bradford assay to predict the total proteins that may be produced and affect the phage/AgNPs interaction (supplementary data). Therefore, the phage stock was centrifugated at medium speed (15,300× *g* for 1 h at 4 °C) in order to reduce the amount of these proteins as much as possible by precipitating the phage and discarding the supernatant that contain most of these proteins.

### 2.8. MIC and MBC

As long as the concentration of AgNPs is higher than 23 µg/mL, no bacterial growth was observed after 24 h. Therefore, the minimum concentration of AgNPs that was able to inhibit the growth of *S.* Enteritidis WT (Platten) is 23 µg/mL and it is the same concentration that was needed to kill the bacteria. This concentration is higher than the concentration that was used by Shimaa et al. who reported that the values of both MIC and MBC were equal to 16 µg/mL when they used it against *S.* Enteritidis bacteria [62]. However, our reported concentration was significantly lower than the concentration used by Ragaa et al. who found that the MIC was 1 mg/mL against *S. enterica* subsp. *salamae* bacterium [63]. We also studied the synergistic effect of both phage ZCSE2 and AgNPs to inhibit the growth of *S.* Enteritidis WT (Platten). For instance, using 0.5× MIC AgNPs with different MOIs of ZCSE2 from 1 to 0.01 showed an inhibitory effect. To the best of our knowledge, this synergetic effect is the first to be reported and proposed a novel approach to control pathogenic bacteria. The applications of nanoparticles to control MRD bacteria opens the door for using it as an alternative to antibiotics. This study is the first to discuss the synergy between biosynthesized sub-lethal dose of AgNPs and phage. Stressing the bacteria under the effect of the sub-lethal dose of AgNPs enabled them to be lysed easily by phage [64] even at low concentrations. From the pharmacokinetic aspect, the high phage dosages will increase its possibility to be detected by the immune system and increase the chance of its clearance from the human/animal body [65]. By optimizing the combination between phage therapy and AgNPs, the dosses of both will be minimized and consequently limit the inflammatory immune responses.

### 2.9. Time-Killing Curve

**Figure 6:**
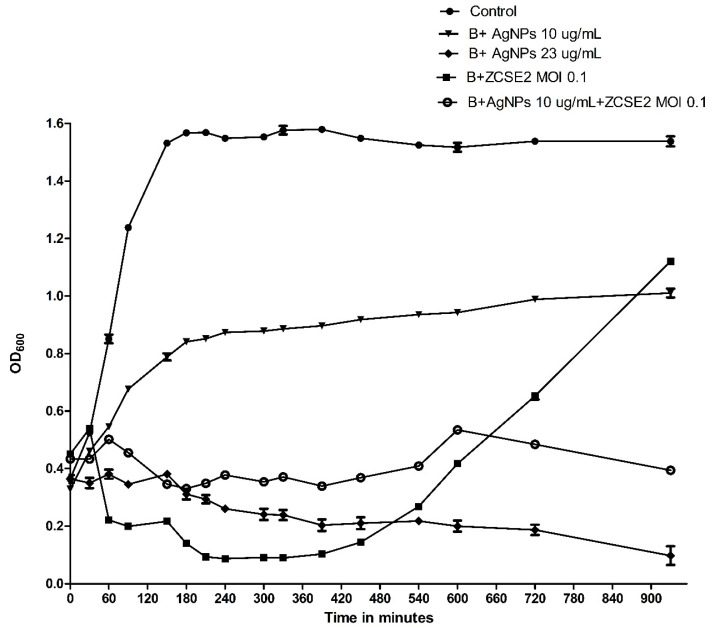
Illustrates the Time-Killing curve of S. Enteritidis *WT (Platten)* with 1× MBC of AgNPs, phage MOI 0.1, AgNPs of final concentration of 10 µg/mL, and mixture of AgNPs of final concentration of 10 µg/mL and phage MOI 0.1.

An effective quantitative analysis was conducted to study the pharmacodynamics of AgNPs, in which the time-killing curves for bacteria were measured as a change in the optical density for λ = 600 (OD_600_) [66]. At the first 360 min (6 h), the curves showed that AgNPs 10 µg/mL had the lowest effect to reduce the bacterial growth, followed by the combination of AgNPs 10 µg/mL and ZCSE2 at MOI of 0.1, then AgNPs 23 µg/mL, while the highest effect was observed with the treatment with ZCSE2 at MOI of 0.1. However, a secondary bacterial growth was observed after the first 6h of infection with the treatment of phage alone due to bacterial resistance. On the other hand, phage ZCSE2 at MOI of 0.1 in combination with AgNPs 10 µg/mL were able to inhibit the bacterial growth after 930 min of the treatment (Figure 6). These results showed the significant difference between using phage alone and using it in combination with biosynthesized AgNPs. As a result, the data indicated that phage ZCSE2 alone at MOI 0.1 was able to reduce the bacterial intensity by 1.5 and 0.416 OD_600_ compared to the control after 330 and 930 min (*p* < 0.001), respectively. Nonetheless, the AgNPs alone reduced the bacterial growth by 0.691 and 0.527 OD_600_ (*p* < 0.001) after 330 and 930 min of the experiment, respectively. On the other hand, the mixture of phage ZCSE2 at MOI 0.1 and 0.4× MIC of AgNPs displayed a reduction of 1.205 and 1.14 OD_600_ compared to the control (*p* < 0.001) after 330 and 930 min of the experiment, respectively. The most interesting observation is that bacterial persistence occurs when the phage was used alone, while it was defeated when the phage was used in combination with AgNPs. Overall, there is a significant inhibitory effect when phage ZCSE2 at MOI of 0.1 combined with AgNPs in a sublethal concentration of 10 µg/mL in comparison to using AgNPs with the same concentration alone or the phage with the same MOI alone. Different experiments support these findings in supplementary data.

## 3. Materials and Methods

### 3.1. Preparation of Propolis

The propolis we used in this experiment was collected from the beehive and weighted as 0.5 g then grounded coarsely by mortar and pestle. The powder was suspended in 50 mL 80% ethanol then placed at 80 °C for 4 h before we store the solution at 4 °C overnight. The solution was filtered through 0.45 µm pore membranes filter (Steradisc, Kurabo Co., Ltd., Osaka, Japan) to remove any suspended particles then stored at −20 °C.

### 3.2. Biosynthesis of AgNPs

The process of biosynthesis of AgNPs was done by preparing 10 mL of 1 mM silver nitrate (Techno pharmachem, India) using deionized water. Exactly, 8.5 mg of silver nitrate was added to 10 mL of deionized water in a beaker and the beaker was covered without any exposure to the light. Exactly, 10 mL of 3% of pre-prepared propolis was added carefully to the silver nitrate solution. Then, the solution was left for 5 h on the hot plate with a magnetic stirrer at different temperatures (25, 55, 85, and 105 °C) to form the nanoparticle. The nanoparticle formation was confirmed by color change (brown) as a first step.

### 3.3. Characterization of AgNPs

#### 3.3.1. UV–Vis Spectroscopy

The formation of AgNPs coated by propolis was characterized by the spectrophotometer (Jenway 7200 visible spectrophotometer) in the range of 340–800 nm. The resulted nanoparticles were diluted 10 folds using deionized water then placed in the cuvette, and the spectrum was measured.

#### 3.3.2. FTIR Analysis

To detect product formation as a result of the interaction among biomolecules found in the propolis and nano silver particles, the bonds were analyzed by the FTIR spectrum of the biosynthesized AgNPs (Agilent system Cary 630 FTIR model)in the range of 4000–400 cm^−1^ at room temperature, as previously described [67].

#### 3.3.3. TEM and Zeta Potential

The size and shape of the biosynthesized AgNPs were investigated by using TEM at the National Research Center (Cairo, Egypt). The produced AgNPs were put on the copper grids and left to dry. Then, the sample was placed in TEM (JEOL 1230). The Image J 1.8v program was used to measure the sizes and perform the magnification. For the determination of the zeta potential of the AgNPs, Zetasizer (Nano ZS, Malvern, UK) was used and the raw data were analyzed by Zetasizer software. The results from Zetasizer were obtained after diluting the sample by 100 folds with deionized water. The zeta potential of AgNPs was examined at Nawah Scientific Company.

### 3.4. Antibacterial Effect of AgNPs

#### 3.4.1. Bacterial Culture

This work was done on *Salmonella* Enteritidis WT (Platten) (S. Enteritidis) that is a gift from The University of Nottingham (United Kingdom). Stocks were maintained in 20% (*v*/*v*) glycerol at −80 °C until needed. Bacterial strains were grown on Tryptone Soya Agar (TSA; Oxoid, UK) overnight at 37 °C. The antibiotic sensitivity test was conducted on this bacterium before and the bacterium was found to be resistant to eight different classes of antibiotics “multidrug resistant bacterium” [6,68].

#### 3.4.2. MIC and MBC of AgNps

To determine the MIC, a microbroth dilution method was conducted by following the procedure described by Prashik et al. [69] by evaluating the visible growth of *S.* Enteritidis in the Tryptone Soya Broth (TSB; Oxoid, UK) with some modifications. Briefly, *S.* Enteritidis was grown on TSA plates. Then, fresh colonies were harvested to be inoculated in the TSB, which incubated overnight at 37 °C. Ten µL of bacteria was added to 90 µL of clear TSB and AgNPs. The AgNPs was adjusted to be diluted by two folds in each well with different concentration from 92 µg/mL to 5.25 µg/mL. After 24 h of incubation, the MIC was determined where the clear well with the lowest AgNPs concentration was observed. In each clear well, 10 µL of TSB was withdrawn and added to fresh liquid media and incubated for another 24 h. After the incubation, the clear well with the lowest concentration of AgNPs was considered as MBC.

#### 3.4.3. Antibacterial Effect of AgNPs Using Disk and Well Diffusion and Direct Spotting

The antibacterial effect of the AgNPs on *Salmonella* was determined by three different methods. First, to predict if the AgNPs have antibacterial activity, direct spotting was used according to Baldi et al. [70]. Briefly, different concentrations of AgNPs and AgNO_3_ (184 µg/mL, 92 µg/mL, 46 µg/mL, 23 µg/mL, 11.5 µg/mL, 5.75 µg/mL) were prepared and directly spotted on an overlay of *S.* Enteritidis on TSA plate. The second method that was used is disc diffusion according to Ajitha et al. with slight modifications [71]. Briefly, a day culture of the bacteria was prepared by adding a single colony to 500 µL TSB and incubated at 37 °C for 2 h. Then, the bacterial culture was swapped on TSA plates and sterile disks were added directly on the surface of TSA plates that have the bacterial culture. Then, the serial dilutions of NPs 10 µL were spotted on the disks. The third method that was used is the well diffusion method according to Gavade et al. [31]. Briefly, different wells were formed in TSA plates that have the bacterial culture, then 20 µL of different concentrations of AgNPs and AgNO_3_ were added. Propolis at two different concentrations (3% and 1.5%) were also used throughout the experiment as a negative control. The inhibition zones were assessed by measuring the diameters of the areas that show no bacterial growth.

### 3.5. Phage Combination with AgNPs

#### 3.5.1. Phage Stability with AgNPs

The *Myoviridae* phage ZCSE2 with ID MK673511 was used in combination with AgNPs to investigate possible synergetic effects. To examine the stability of phage ZCSE2, we added 100 µL of phage at 10^6^ PFU/mL to an Eppendorf containing 100 µL of AgNPs (184 µg/mL) to reach to 92 µg/mL of AgNPs as a final concentration and incubated it for four hours at 37 °C in shaking incubator. The phage titer was determined before and after incubation to test its stability by double-agar overlay plaque assays [32]. Briefly, 100 µL of the bacterial culture was added to 4 mL of molten 0.7% (Bacto) top agar (≈55 °C), and poured on the top of TSA plates. After 15 min, 10 µL of 10-fold serial-diluted phage and phage mixed with biosynthesis AgNPs were spotted on the bacterial lawn. The plates were left until the spots were dried and incubated upside down overnight at 37 °C.

#### 3.5.2. MIC for Phage and AgNPs

The MIC was conducted as described above with different phage titers from 10^6^ PFU/mL to lower than 10^1^ PFU with 0.5× MIC of AgNPs and in another experiment without AgNPs to measure the MIC for the phage alone. The bacterial initial concentration to measure the MIC was 1.5 × 10^7^ CFU/mL to achieve a wide range of MOIs, from 0.1 to 0.000001.

#### 3.5.3. In Vitro Time-Kill Assay

A cuvette containing 1 mL of *S.* Enteritidis in TSB at 0.35 OD_600_ was used as a positive control. Another four cuvettes; one containing the bacteria with the phage at MOI of 0.1, one containing the bacteria and AgNPs with final concentration of 10 µg/mL, one containing the bacteria and AgNPs with final concentration of 23 µg/mL, and the last one containing phage ZCSE2 MOI 0.1, AgNPs with final concentration of 10 µg/mL and bacteria were used. In addition, a negative control (fresh TSB without bacterial growth) was used as a blank at the time point zero. All cuvettes were incubated at 37 °C with gentle shaking for 930 min. During the incubation period the samples were analyzed by measuring the OD_600_ at defined time points (0, 30, 60, 90, 150, 180, 210, 240, 300, 330, 390, 450, 540, 600, 720, and 930 min).

## 4. Conclusions

This study provides a novel approach by using a combination of phage and nanoparticles as an alternative to antibiotics to get the maximum synergistic effect to control pathogenic bacteria. The biosynthesized AgNPs were produced from silver nitrate and propolis extract, in which they were characterized through a color change, UV–Vis spectrum, Zeta potential, FTIR, and TEM. The antibacterial effect of AgNPs alone and in combination with ZCSE2 against *Salmonella* was evaluated by measuring MIC, MBC, time-killing curve, bacterial survival and reduction. The data showed that the combination of AgNPs and phage ZCSE2 reduced the bacterial growth significantly in comparison with other treatments and this was clear when the bacterial turbidity after 15.5 h showed a high reduction in OD_600_ for the treatment of both phage and AgNPs in comparison to the phage treatment. Our results suggest that the combination of phage and nanoparticles has a potential for phage applications to control bacterial infections.

## Figures and Tables

**Table 1:** Zone of inhibition obtained though the direct spotting, disk and well diffusion tests.

Serial Dilution	Dimeter (mm)		
	Direct Spotting	Disk Diffusion	Well Diffusion
3% and 1.5% Propolis extract	without antibacterial effect	0	0
184 µ/mL of AgNPs	antibacterial effect	11	12
92 µg/mL of AgNPs	antibacterial effect	10	10
46 µg/mL of AgNPs	antibacterial effect	8	10
23 µg/mL of AgNPs	antibacterial effect	9	7
11.5 µg/mL of AgNPs	without antibacterial effect	8	6~7

## Supplementary Materials

The following are available online at https://www.mdpi.com/article/10.3390/antibiotics10060678/s1, Figure S1: Shows the effect of using ZCSE2, AgNPs, and the mixture of both of them on bacterial growth, Figure S2: Shows Time-Killing curve of S. Enteritidis with phage at MOI 0.03, AgNPs of final concentration of 10 µg/mL, and mixture of AgNPs of final concentration of 10 µg/mL and phage MOI 0.03, Figure S3: Shows the comparison between the inhibition zone of AgNPs in disc diffusion, Figure S4: Shows the zeta potential distribution for biosynthesized AgNPs with a single peak at 22.2mV, Figure S5: Represents FTIR spectrum of biosynthesized AgNPs, Table S1: Shows the values of released proteins after using AgNPs, phage, and the combination of both of them, Table S2: Shows the inhibition zones resulting from direct spotting, disk and well diffusion tests for AgNO_3_ solution.
