# Supplementary material for: The Synergistic Effect of Biosynthesized Silver Nanoparticles and Phage ZCSE2 as a Novel Approach to Combat Multidrug-Resistant Salmonella enterica
Source: Antibiotics (Basel). 2021 Jun 5;10(6):678. doi: 10.3390/antibiotics10060678 (PMC8228988; doi:10.3390/antibiotics10060678)
Supplement: Supplementary file 1 [file antibiotics-10-00678-s001.zip › antibiotics-1186010-supplementary.pdf]

Supplementary Materials

1. Results and Discussion

1.1. Bradford assay

The results of the protein leakage profile indicated that there was no bacterial protein release detection observed upon AgNPs treatment, whereas a low leakage was detected when the combination of phage and AgNPs was used. For the AgNPs treatment, we expect that the small size of nanoparticles allowed them to penetrate through the bacterial cell wall and act as free Ag^{+} ions leading to reactive oxidative stress (ROS), as described in previous work [1]. However, to confirm that this is due to ROS, further experiments are required. The leakage of protein in the phage treatment is due to bacterial lysis. It was clear that the concentration of protein varies according to the used MOIs, as indicated in Table S1. At all MOIs, the combination of phages and AgNPs resulted in a relatively lower protein leakage, which is considered a good advantage for in vivo experiments and clinical trials to reduce any possible immune responses due to phage treatment.

Table S1. Shows the values of released proteins after using AgNPs, phage, and the combination of both of them. .

Treatment	The mean absorbance value at OD_{595}	Estimated released proteins in µg/mL
Control	0	0
Bacteria+ AgNPs 11.5 µg/mL as final concentration	0.024	NA
Bacteria + ZCSE2 MOI 1	0	0
Bacteria + ZCSE2 MOI 1+ AgNPs 11.5 µg/mL as final concentration	0	0
Bacteria + ZCSE2 MOI 0.1	0.156	143
Bacteria + ZCSE2 MOI 0.1+ AgNPs 11.5 µg/mL as final concentration	0.058	NA
Bacteria + ZCSE2 MOI 0.0001	0.163	157
Bacteria + ZCSE2 MOI 0.0001+ AgNPs 11.5 µg/mL as final concentration	0.098	33.8
Bacteria + ZCSE2 MOI 0.00001	0.203	232
Bacteria+ ZCSE2 MOI 0.00001+ AgNPs 11.5 µg/mL as final concentration	0.0997	36.9

1.2. Bacterial challenge and turbidity assay

Another experiment was conducted to predict the efficiency of using a combination of phage and AgNPs versus using the AgNPs or phage ZCSE2 alone. The efficacy of the treatments was evaluated by counting the CFU/ml. Both treatments showed a reduction in the viable count compared to the control ($p < 0.001$; Figure S1 A and B), at the experimental end point of 180 min. Within 180 min, phage ZCSE2 presented a high reduction rate of bacteria in comparison to the combination of AgNPs and phage ZCSE2. However, after 24 h of incubation, the combination of AgNPs and phage ZCSE2 showed a higher level of bacterial reduction than using both of them separately (Figure S1 C-F). This may be due to the presence of persistent bacteria that emerged over the long period of incubation [2,3]. In the treatment of phage ZCSE2 and AgNPs combination at both MOIs (1 or 0.1 with 0.5xMIC of AgNPs) at OD_{600} and after 24h of incubation (Figure S1 E, F , C and D), it was clear that the combination of both phage and AgNPs managed to control the bacterial growth significantly over time. This work highlights the possibility of using a combination of phage and AgNPs as an alternative to control *Salmonella*.

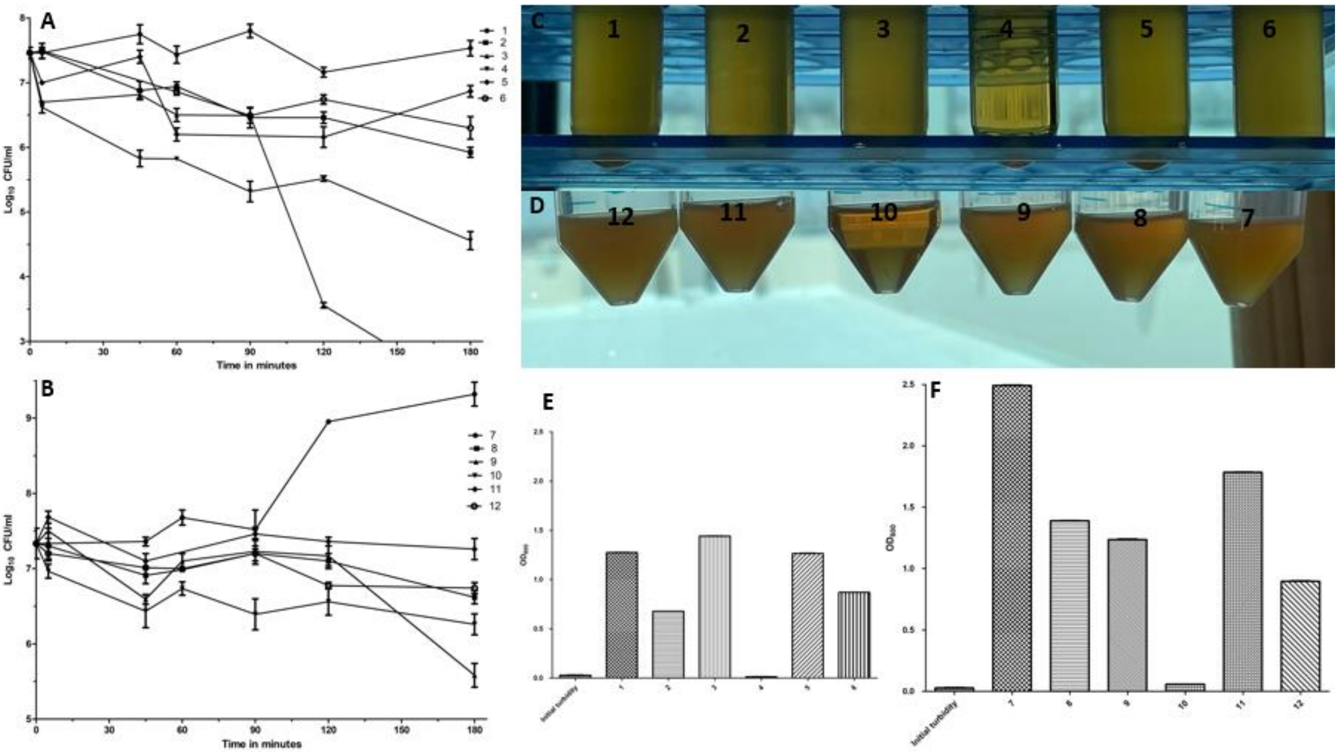

Figure S1. Shows the effect of using ZCSE2, AgNPs, and the mixture of both of them on bacterial growth. Figures C and D: 1 and 7) Bacteria without treatment. 2 and 8) Bacteria with AgNPs final concentration of 11.5 µg/mL. 3, 5,9, and 11) Bacteria with phage alone at MOI of 1, 0.00001, 0.1, and 0.0001, respectively. 4, 6, 10, and 12) bacteria with AgNPs final concentration of 11.5 µg/mL and phage at MOI of 1, 0.00001, 0.1, and 0.0001, respectively. Graphs A and B represents the bacterial survival estimated by counting CFU over 180 min. C and D) The bacterial turbidity after incubation of bacteria in graphs A and B, respectively, in sterile glass test tubes and falcons for 24h. E and F). The OD_{600} data for bacteria are shown in Figures C and D respectively.

1.3. Time-Killing curve

At MOI 0.1, the ZCSE2, in combination with AgNPs at final concentration of 10 µg/ml, showed the ability to control the bacterial growth after 930 min better than the individual application of phage or 10 µg/ml AgNPs as final concentration. Another experiment was conducted with lower MOI to confirm this conclusion. The secondary bacterial growth appeared after 330 min and this highlights the importance of using the combination of phage and AgNPs to see the synergistic effect of both of them (Figure S2).

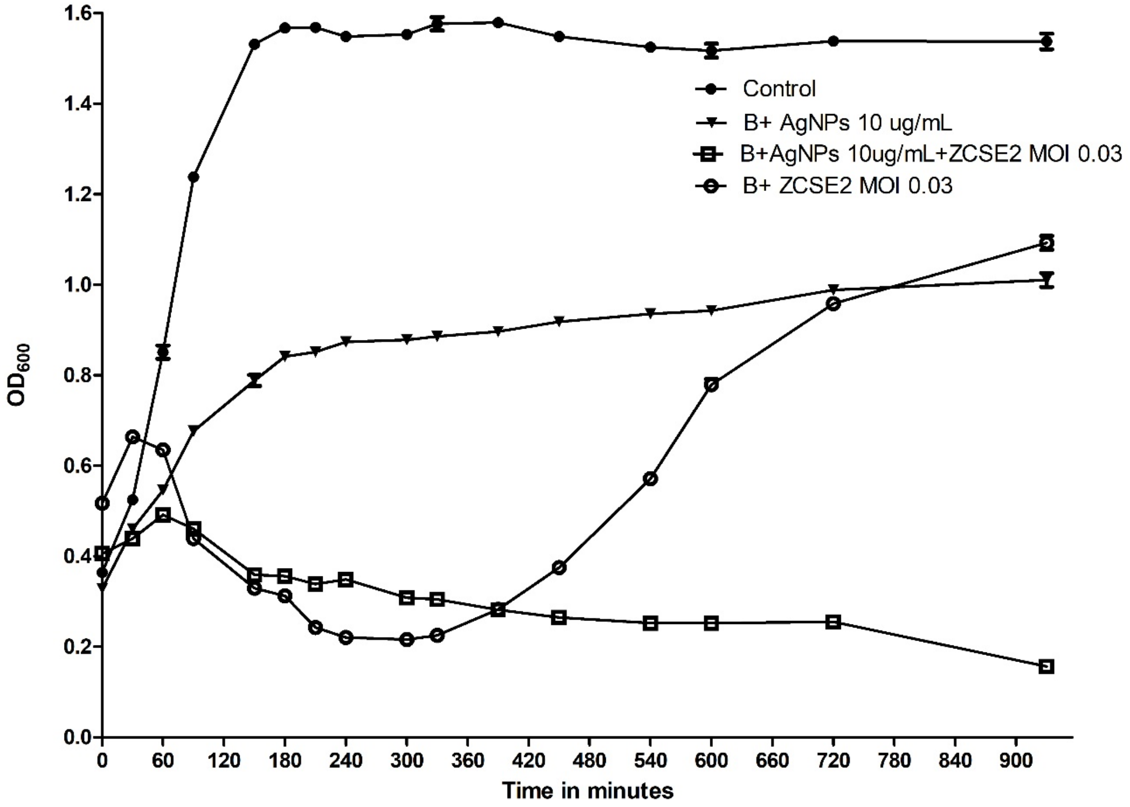

Figure S2. Shows Time-Killing curve of S. Enteritidis with phage at MOI 0.03, AgNPs of final concentration of 10 µg/ml, and mixture of AgNPs of final concentration of 10 µg/ml and phage MOI 0.03.

1.4. Disc and well diffusion methods

Table S2. Shows the inhibition zones resulting from direct spotting, disk and well diffusion tests for $AgNO_3$ solution.

Serial Dilution	Dimeter (mm)		
	**Direct spotting**	**Disk Diffusion**	**Well Diffusion**
184 µ/ml of $AgNO_3$	antibacterial effect	11	13
92 µg/ml of $AgNO_3$	antibacterial effect	11	10
46 µg/ml of $AgNO_3$	antibacterial effect	9	8
23 µg/ml of $AgNO_3$	antibacterial effect	9	6~7
11.5 µg/ml of $AgNO_3$	without antibacterial effect	0	0

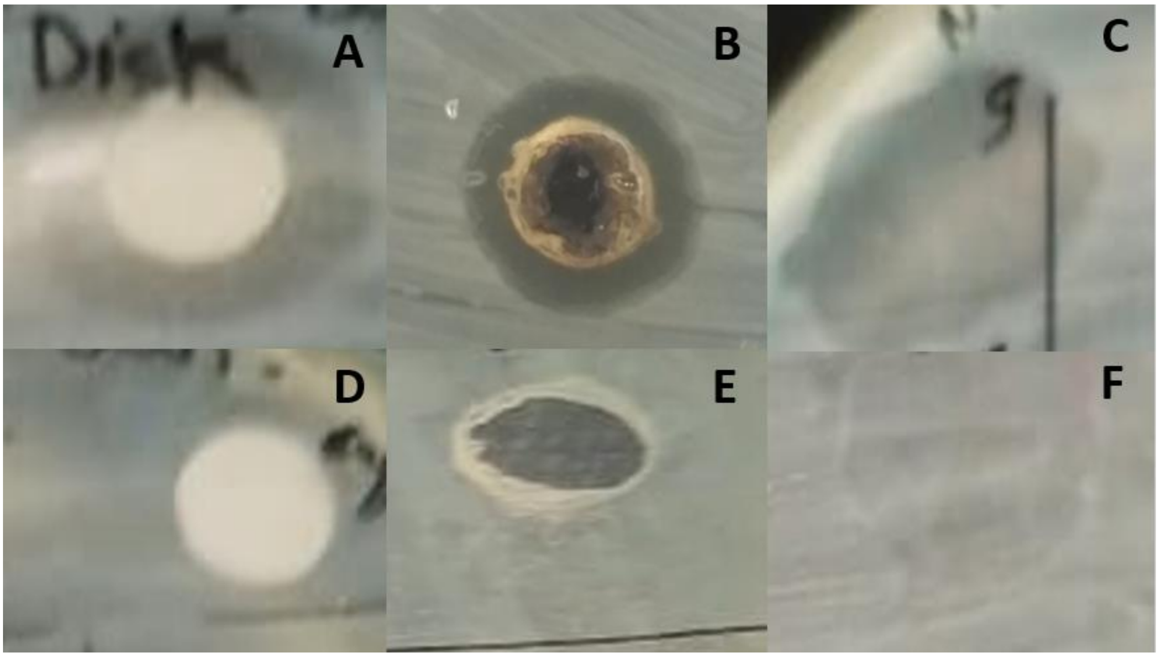

Figure S3. Shows the comparison between the inhibition zone of AgNPs in disc diffusion, well diffusion and direct spotting (A, B, and C) respectively, versus the non-effect of 3% of propolis extract against S. Enteritidis WT platen in disc diffusion, well diffusion and direct spotting (D, E, and F), respectively.

1.5. Zeta potential

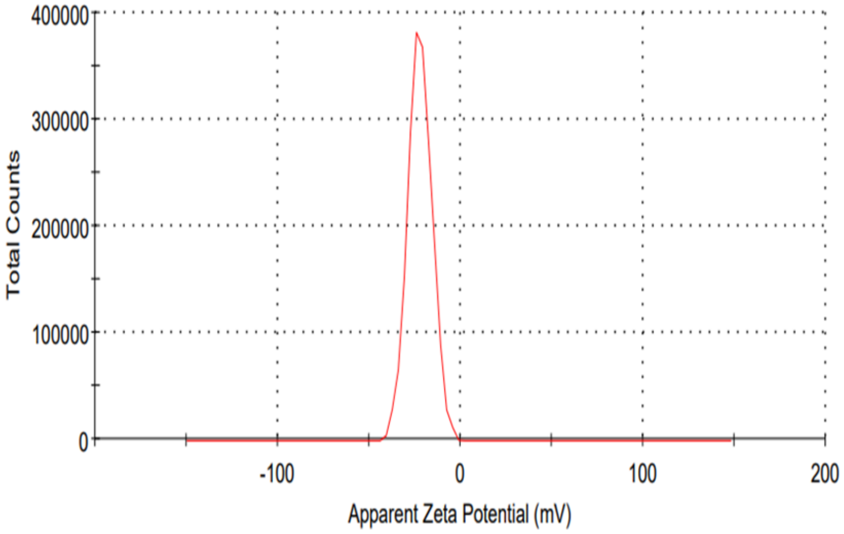

Figure S4. Shows the zeta potential distribution for biosynthesized AgNPs with a single peak at 22.2mV.

1.6. FTIR Analysis

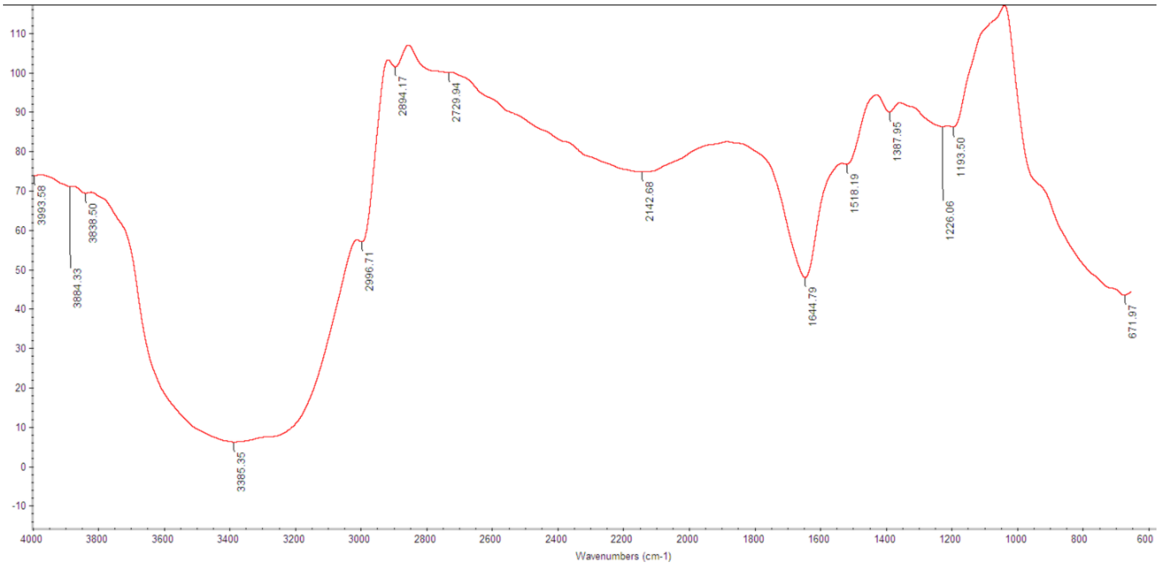

Figure S5. Represents FTIR spectrum of biosynthesized AgNPs.

2. Material and Methods

2.1. Protein leakage analysis

To quantify the leaking protein upon treatment with nanoparticles, the Bradford assay was followed [4]. The experiment aims to compare the protein leakage of three treatments that target *S. enterica*: AgNPs only, phage ZCSE2 only and the combination of both AgNPs and ZCSE2 at different MOIs. Ten sets were prepared (control, 11.5 µg/mL final concentration of AgNPs, ZCSE2 (MOI 1, 0.1 0.0001 and 0.00001), and 11.5 µg/mL AgNPs in combination with ZCSE2 (MOI 1, 0.1, 0.0001 and 0.00001)). Bacteria with initial titer of 10^7 were used (OD_{600} of 0.02~0.03). The ten sets were incubated for 24 h. Then, 1 ml of each was collected and centrifuged at 6000 rpm, for 15 min. Furthermore, 500 µL of each supernatant was added to 500 µL of Coomassie brilliant blue stain of the Bradford assay and incubated for 10 min, then the OD_{595} was measured. The standard curve was made using Bovine Serum albumin.

2.2. Bacterial challenge and bacterial turbidity assay

Fresh viable *S.* Enteritidis was used to inoculate thirteen tubes containing 5 mL of TSB to reach a final bacterial concentration of $2x10^7$ CFU/ml and biosynthesised AgNPs at a final concentration of 11.5 µg/ml, 5 mL of TSB with phage ZCSE2 at different MOIs of (1, 0.1, 0.0001 and 0.00001), 5 mL of TSB to reach a final bacterial concentration of $2x10^7$ CFU/ml with biosynthesized AgNPs at a final concentration of 11.5 µg/ml and phage ZCSE2 at different MOIs of (1, 0.1, 0.0001 and 0.00001), and 5 mL of TSB to reach a final bacterial concentration of $2x10^7$ CFU/ml. A tube with TSB without bacteria was used as a negative control. At different time intervals (0, 5, 45, 60, 90, 120, and 180 min), 100 µl of the suspension-containing bacteria from each tube was withdrawn and serially diluted in TSB. Ten µl of each dilution were spotted on TSA plates. After drying the spots, the plates were incubated upside down at 37°C overnight, then the number of colonies were counted on the plates. After 24 h of ten tubes incubation at 37°C, 1 mL of each tube was withdrawn and the absorbance was measured at OD_{600}.

2.3. In Vitro Time Kill Assay

A cuvette containing 1 mL of *S.* Enteritidis in TSB at 0.35 OD_{600} was used as a positive control. Another four cuvettes were used as follows; one contained the bacteria with the phage at MOI of 0.1, one contained the bacteria and AgNPs with a final concentration of 10 µg/ml, one containing the bacteria and AgNPs with final concentration of 23 µg/ml,

and one containing phage ZCSE2 at MOI 0.03, AgNPs with final concentration of 10 μg/ml and bacteria. In addition, a negative control that consists of fresh TSB without infection was used as a blank at the time point zero. All cuvettes were incubated at 37 °C with gentle shaking for 930 min. During the incubation period, the samples were analyzed by measuring the OD_{600} at defined time points (0, 30, 60, 90, 150, 180, 210, 240, 300, 330, 390, 450, 540, 600, 720, and 930 min).

References

1. Qing, Y.; Cheng, L.; Li, R.; Liu, G.; Zhang, Y.; Tang, X.; Wang, J.; Liu, H.; Qin, Y. Potential antibacterial mechanism of silver nanoparticles and the optimization of orthopedic implants by advanced modification technologies. *Int. J. Nanomedicine* **2018**, *13*, 3311–3327.
2. Patra, P.; Klumpp, S. Population dynamics of bacterial persistence. *PLoS ONE* **2013**, *8*, e62814.
3. Abdelsattar, A.S.; Dawoud, A.; Makky, S.; Nofal, R.; Aziz, R.K.; El-Shibiny, A. Bacteriophages: From isolation to application. *Curr. Pharm. Biotechnol.* **2021**, doi:10.2174/1389201022666210426092002.
4. He, F. Bradford Protein Assay. *BIO-PROTOCOL* **2011**, *1*, doi:10.21769/bioprotoc.45.
